# Personality and Its Influence on Pain Sensitivity Based on Different Hormonal Responses to Individual vs. Group Exercise Styles

**DOI:** 10.3390/life15020222

**Published:** 2025-02-02

**Authors:** Aya Nakae, Hani M. Bu-Omer, Chie Kishimoto, Wei-Chuan Chang, Hidenobu Sumioka

**Affiliations:** 1 Presence Media Research Group, Hiroshi Ishiguro Laboratories, Deep Interaction Laboratory Group, Advanced Telecommunications Research Institute International (ATR), 2-2-2 Hikaridai, Seika-Cho, Soraku-Gun, Kyoto 619-0288, Japan; hbuomer@atr.jp (H.M.B.-O.); kishimotochie@atr.jp (C.K.); chou@atr.jp (W.-C.C.); sumioka@atr.jp (H.S.); 2 Global Center for Medical Engineering and Informatics, Graduate School of Medicine, Osaka University, 2-2 Yamadaoka, Osaka 565-0871, Japan; 3 Laboratory of Science & Innovation for Pain, Graduate School of Frontier Biosciences, Osaka University, 2-2 Yamadaoka, Osaka 565-0871, Japan

**Keywords:** personality differences, pain sensitivity, cortisol, growth hormone, KAATSU training, group training, NEO-PI-R, hormonal changes

## Abstract

Individual differences in pain sensitivity are thought to relate to personality traits, but the underlying mechanisms remain unclear. Exercise influences hormonal secretion via the hypothalamic–pituitary system, which may link personality, hormonal responses, and pain perception. This study investigated these relationships in 14 healthy participants (3 females, 11 males, aged 20–50 years, mean 28 ± 9.25 years). Participants rated thermal pain stimuli and completed the NEO Personality Inventory-Revised (NEO-PI-R) to identify their personality. Each participant engaged in personal and group training sessions, with blood samples collected to measure cortisol, growth hormone, and other indicators. Participants were clustered into cortisol hypersecretors and hyposecretors based on their hormonal response. Hypersecretors exhibited significantly lower neuroticism scores and pain ratings than hyposecretors. These findings suggest a potential association between cortisol responsiveness during exercise, neuroticism, and pain sensitivity. This study highlights potential links between personality traits and reactive hormonal patterns, offering insights into the psychophysiological mechanisms underlying pain expression.

## 1. Introduction

Research indicates a connection between pain sensitivity and neurotic personality traits, yet evidence remains limited and fragmented. A relationship exists between aversive responses to daily sensations and traits such as neuroticism, openness, and extraversion, which contribute to daily pain sensitivity [1]. Specific aspects of personality, such as impulsiveness (a facet of neuroticism) and excitement-seeking (a facet of extraversion), have been linked to cold pain intensity [2]. Furthermore, psychological tools like the Five-Factor Inventory for personality assessment and the Pain Catastrophizing Scale have been proposed as potential tools for identifying personality traits associated with high pain sensitivity [3]. However, the interplay between pain sensitivity, personality traits, and hormonal secretion characteristics, particularly under the influence of exercise, has not been thoroughly examined.

Exercise-induced hypoalgesia (EIH), a phenomenon where pain sensitivity decreases after exercise, has been consistently observed in healthy populations. Both aerobic and resistance exercises reduce pain sensitivity across various modalities, including pressure, thermal, and electrocutaneous pain thresholds [4]. Variability in EIH outcomes, however, has been noted, with some individuals experiencing less pronounced or even adverse effects [5,6,7]. This variability may stem from differences in personality traits and hormonal reactivity, underscoring the need for further investigation.

The mechanisms of EIH have been extensively studied in animal models, focusing on the roles of inflammatory cytokines, neurotrophins, neurotransmitters, endogenous opioids, and histone acetylation at different sites in the peripheral nervous system, including injured peripheral nerves, dorsal root ganglia, and the spinal dorsal horn [8]. Exercise also influences pain perception through the endogenous opioid system, releasing endorphins with effects similar to morphine, contributing to pain relief [9]. Therefore, exercise is considered a promising approach for predicting individual differences in pain perception via hormone secretion.

Exercise has also been shown to improve mental health by alleviating mood and reducing stress and depression, which are often associated with chronic pain conditions [7]. However, exercise is not universally effective in treating depression, partly due to adherence issues and individual differences in responsiveness, including both physical and motivational factors [5,6]. Patients with chronic pain often exhibit inconsistent or adverse responses to exercise rehabilitation, with some experiencing increased pain sensitivity [7]. These outcomes highlight the need to explore how individual factors, such as personality traits and hormonal reactivity, influence EIH.

The Big Five personality traits model identifies five key dimensions of personality: openness, conscientiousness, extraversion, agreeableness, and neuroticism [10]. These traits account for much of the variation in personality descriptions and are consistent across cultures and age groups. Each trait plays a distinct role in emotional regulation, stress reactivity, and coping mechanisms, which are particularly relevant to mood, pain perception, and pain response. Neuroticism, characterized by emotional instability and a tendency toward negative affect, is often associated with heightened pain sensitivity and lower pain thresholds [3]. Extraversion, defined by sociability and positive affect, may enhance coping mechanisms and reduce perceived pain intensity [2]. Openness, reflecting creativity and curiosity, has been linked to adaptive responses to novel pain experiences [11]. Agreeableness, associated with empathy and cooperation, may influence interpersonal coping strategies in pain management [12], while conscientiousness, defined by self-discipline and organization, can improve adherence to pain interventions and reduce stress-related reactivity [13]. Understanding these traits and their influence on physiological and psychological responses provides a foundation for exploring individual differences in hormonal reactions and pain modulation.

Hormonal responses, particularly those mediated by the hypothalamic–pituitary–adrenal (HPA) axis, play a central role in stress reactivity and pain perception. Cortisol, a key HPA-axis hormone, influences stress-induced changes in pain thresholds by modulating physiological and emotional responses [14]. Neuroticism, characterized by heightened emotional reactivity, has been associated with increased cortisol levels under stress, potentially lowering pain thresholds and altering stress-coping mechanisms [2,3]. While existing studies have demonstrated the relationship between neuroticism and cortisol reactivity, the combined effects of personality traits, exercise conditions, and hormonal profiles on pain modulation remain poorly understood.

Although gender and age effects were not explicitly analyzed in this study due to the small sample size, the existing literature highlights their importance in shaping pain perception and hormonal responses. Women often exhibit greater pain sensitivity than men, which may be partially attributed to hormonal fluctuations, particularly estrogen levels, affecting the HPA axis [15]. Age-related changes in the HPA axis, including altered cortisol reactivity and reduced adaptability to stress, are well-documented and may influence pain sensitivity and stress responses in older populations [16,17]. These factors could modulate the relationships between personality traits, hormonal responses, and pain sensitivity, which are evaluated using the NEO-PI-R, POMS-2, and other scales in this study.

This exploratory study aimed to investigate the relationships between personality traits, hormonal secretion—focusing on the HPA axis—and individual differences in pain perception. Specifically, the study hypothesized that personality traits, such as neuroticism, may influence or be associated with individuals’ hormonal responses to exercise (e.g., cortisol secretion), which in turn modulate pain sensitivity. By examining hormonal and pain expression changes before and after group and personal training exercises, which represent contrasting social and motivational contexts, this study seeks to identify patterns linking personality, hormonal reactivity, and pain perception.

## 2. Methods

**Figure 1:**
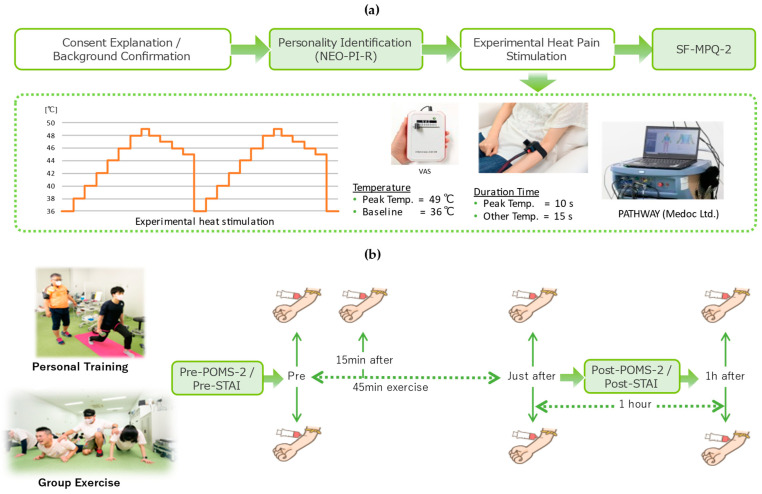
Study design: (**a**) Identification of personality traits using the NEO-PI-R and assessment of pain expression with thermal pain stimulation and the Short-Form McGill Pain Questionnaire-2 (SF-MPQ-2). (**b**) Exercise tasks (group training and personal training sessions) and the corresponding timing of blood collection and questionnaires. Green arrows indicate the sequence of events and the timing of blood sampling and questionnaire administration (Pre, 15 min after, Just after, and 1 h after the exercise sessions).

This study complied with the Declaration of Helsinki and received approval from the Ethics Review Committee of the Graduate School of Frontier Biosciences, Osaka University (Application Number: FBS2020-13, Approval date: 13 January 2021). All participants provided written consent prior to enrollment. The study consisted of two stages/experiments. The first stage involved assessing participants’ personality traits and subjective pain expression through relevant questionnaires. The second stage comprised two exercise sessions conducted on separate days. The type of exercise differed between the two sessions—either group training or personal training. Mood and mental status changes were measured before and after exercise. Hormonal changes were also assessed at different stages of the exercise sessions. The study design is shown in Figure 1, with further details provided in the following subsections.

### 2.1. Participants

Fourteen healthy participants (3 females, 11 males, aged 20–50 years, mean 28 ± 9.25 years) were recruited based on the following criteria: (1) age over 18; (2) no chronic pain; (3) no use of analgesics or psychoneural drugs; and (4) ability to understand and follow study instructions in Japanese. Participants were randomly assigned to start with either group training (half) or personal training. The group training session was held once for all participants together. Thereafter, data of all participants were statistically analyzed.

### 2.2. Personality Assessment

The participants’ personality traits were assessed using the NEO Personality Inventory-Revised (NEO-PI-R) [18], which evaluates five major personality domains: neuroticism, extraversion, openness, agreeableness, and conscientiousness, each with six facets (e.g., anxiety under neuroticism, trust under agreeableness). The questionnaire includes 240 questions answered on a five-point Likert scale from 1 (strongly disagree) to 5 (strongly agree).

### 2.3. Subjective Evaluation of Pain

Participants underwent thermal pain stimulation and rated their experience. Seated comfortably in an armchair in a quiet room at 22–24 °C, they placed their left hand on the chair’s armrest. Heat stimuli were delivered using Pathway Thermal Stimulator [19] with a thermode gently applied to the ventral side of the distal left forearm, which was held for at least 30 s before the stimulus. The heat pattern was identical for all participants, as shown in Figure 1a. After the stimulation, participants completed the Japanese version of the Short-form McGill Pain Questionnaire (SF-MPQ-2) to assess their pain. The SF-MPQ-2 includes 22 items across four subscales: continuous pain, intermittent pain, neuropathic pain, and affective descriptors, providing a detailed evaluation of pain intensity, quality, and emotional impact [20,21].

### 2.4. Training Protocol

Participants attended two types of training sessions. The first was a 45 min group exercise (Circuit Training), including modified high-intensity interval exercises with warm-up and cool-down. Following a brief warm-up, all participants performed lower-body strength exercises (squats, lunges), crunches, push-ups, jumping jacks, and burpees for 1–2 min, with 2–3 min of jogging during rest periods. The instructor provided brief instructions during rest, including how to adjust intensity (e.g., touching the knee to the ground during push-ups). Participants chose their own training intensity.

The second session involved KAATSU training as a personal training style. KAATSU is a low-intensity resistance exercise with restricted blood flow to the muscles, aimed at promoting muscle hypertrophy and strength [22]. Following standard KAATSU protocol, pneumatic cuffs (TD 312 calculating cuff inflator, Hokanson, Bellevue, WA, USA) were applied to the limbs, inflated to 1.5 times the systolic blood pressure (135–186 mmHg) just before the exercise, and remained inflated throughout the session and rest periods, totaling around 8 min. The exercise intensity and protocol were chosen based on evidence supporting enhanced muscle adaptations with blood flow restriction during low-intensity resistance exercise [22].

Notably, the purpose of setting the two exercises was not to study the effects of the type of exercise itself but to assess how individuals with different personalities responded to instruction in a group exercise (with less restrictions to follow the instructor) versus personal training settings.

### 2.5. Blood Samples for Hormone Level Measurements

Participants’ blood samples were taken at three time points: just before the training (baseline), just after the training, and one hour after the end of the training. In the personal training session, participants were requested to provide additional blood samples 15 min after starting the KAATSU training.

The group training session was conducted as a single session with all participants simultaneously, which posed logistical challenges for collecting additional blood samples without disrupting the activity. Collecting a sample during the middle of the session would require participants to leave and return to the activity at different times, potentially affecting the uniformity of the training experience. To ensure consistency and minimize disruptions, blood samples during the group training session were limited to three key time points: pre-exercise, post-exercise, and 1 h after exercise. In contrast, the flexibility of the personal training session allowed for individualized sampling, enabling the collection of an additional sample 15 min after the start of the exercise to capture rapid hormonal changes.

Serum levels of cortisol, dehydroepiandrosterone–sulfate (DHEA-S), growth hormones (GH), and leptin were determined using an enzyme immunoassay (EIA) technique (cortisol: Detect X Cortisol Enzyme Immunoassay Kit, Arbor Assays, Ann Arbor, MI, USA; DHEA-S: DHEA-S ELISA RUO, DRG International, Inc., Springfiled, NJ, USA; GH: Quantikine ELISA Human Growth Hormone Immunoassay, R&D Systems, Inc., Minneapolis, MN, USA; Leptin: Human Leptin (highly sensitive) Assay Kit, Immuno-Biological Laboratories Co., Ltd., Gunma, Japan). The limit of detection was 45.5 pg/mL (0.00455 ug/dL), 0.044 ug/mL, 2.10 pg/mL, and 2.13 pg/mL, for cortisol, DHEA-S, GH, and leptin, respectively; the intra- and inter-assay coefficients of variation were <10% for all assays, except leptin (below 10.2%).

The serum level of L-lactate was determined via a bioluminescent assay (Lactate-Glo^TM^ Assay, Promega Co., Madison, WI, USA). The limit of detection was 200 uM; the intra- and inter-assay coefficient of variation were not provided by the manufacturer.

The acidified plasma level of active ghrelin was determined using Enzyme-Linked Immunosorbent Assays (ELISAs) (Active Ghrelin: Active Ghrelin ELISA kit, LSI Medience Corporation, Tokyo, Japan). The limit of detection was 160 fmol/mL for active ghrelin; the intra- and inter-assay coefficient of variation were not provided by the manufacturer.

### 2.6. Mood and Mental Status Evaluation

The participants’ mood and mental status changes before and after exercises were assessed using the Profile of Mood States 2nd Edition (POMS-2), a tool for assessing transient and distinct mood states [23,24]. The complete version of the POMS-2 comprised 65 questions and T-scores of six mood clusters: anger hostility (AH), confusion bewilderment (CB), depression dejection (DD), fatigue inertia (FI), tension anxiety (TA), and vigor–activity (VA). We determined total mood disturbance (TMD) by summing the T-scores of AH, CB, DD, FI, and TA (negative mood states) and by subtracting the VA T-score (positive mood state). Friendliness (F) was considered separate from the other mood states.

Furthermore, the Spielberger State–Trait Anxiety Scale (STAI) was used to assess anxiety levels. The STAI questionnaire score ranges from 20 to 80 points and is divided into four groups: no (≤20), mild (21–39), moderate (40–59), and severe (60–80) anxiety [25,26]. The participants completed the two questionnaires at the starting and ending points of each training session.

### 2.7. Statistical Analyses

Statistical analyses were performed using JMP^®^, Version 16.0 (SAS Institute Inc., Cary, NC, USA). The participants were categorized into two groups, Type1 (higher cortisol secretors) and Type2 (lower cortisol secretors), based on their cortisol secretion patterns using hierarchical clustering (Ward’s method) [27]. This clustering approach categorized participants according to similarities in their cortisol secretion data across seven measured time points (baseline, 15 min after the start of personal training, post-exercise, and recovery) without relying on predefined cutoff values. This method has been widely applied to identify distinct physiological profiles in previous research [28].

Paired *t*-tests were conducted to compare hormone levels and questionnaire scores between different time points within each exercise condition. Independent *t*-tests were used to compare the means of the two cortisol-based groups (Type1 and Type2) across various variables, including hormonal responses, pain sensitivity scores, and personality traits. Statistical significance was set at *p* < 0.05 for all tests.

The use of *t*-tests in small sample studies is well-supported in the statistical literature, as they are robust to minor deviations from normality and variance equality, especially in exploratory research with balanced group sizes. Studies have demonstrated that *t*-tests perform well under such conditions, making them suitable for this analysis [29,30].

All data are presented as means ± standard error of the mean (SEM) unless otherwise specified. Correlation analyses were performed where appropriate to explore relationships between variables. Figures depicting hormonal and questionnaire results include annotations to indicate statistically significant differences.

## 3. Results

### 3.1. Hormonal Changes: Group vs. Personal Training

#### 3.1.1. Cortisol Levels

In the personal training condition (KAATSU), compared to baseline, serum cortisol levels significantly increased in all intervals (15 min *p* = 0.0101, 95% CI [1.3241, 8.0952]; just after *p* = 0.0002, 95% CI [4.6833, 11.571]; 1 h after *p* = 0.0030, 95% CI [1.6967, 6.6338]). Compared to just after the training, the serum cortisol level significantly decreased an hour after the training (*p* = 0.0025, 95% CI [−7.2684, −0.6554]).

**Figure 2:**
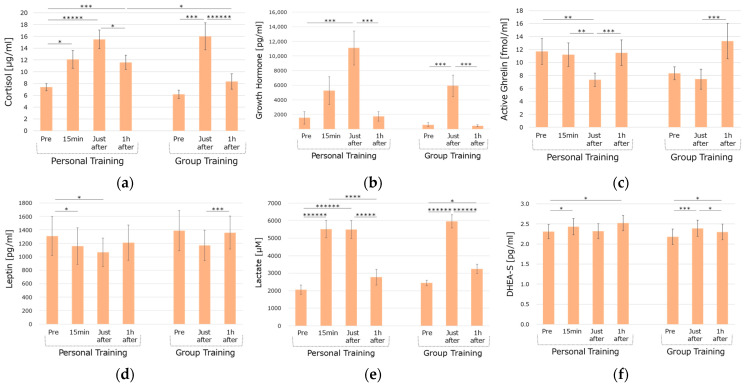
Patterns of hormonal secretion in the two different training conditions. (**a**): cortisol levels, (**b**): GH levels, (**c**): active ghrelin levels, (**d**): leptin levels, (**e**): lactate levels, (**f**): DHEA-S levels. Significant differences are indicated as follows: * *p* < 0.05, ** *p* < 0.01, *** *p* < 0.005, **** *p* < 0.001, ***** *p* < 0.0005, ****** *p* < 0.0001.

In the group (circuit) training condition, compared to baseline, the serum cortisol level significantly increased just after the training (*p* = 0.0014, 95% CI [4.5521, 15.097]). Compared to just after the training, the serum cortisol level significantly decreased an hour after the training (*p* < 0.0001, 95% CI [−10.589, −4.7232]) (Figure 2a).

#### 3.1.2. GH Levels

In the personal training condition, compared to pre-training, the serum GH levels significantly increased just after the training (*p* = 0.0019, 95% CI [4211, 14,843]) and significantly decreased 1 h after the training (*p* = 0.0010, 95% CI [−14,127, 4547]).

In the group training condition, compared to the baseline, the serum GH level significantly increased just after the training (*p* = 0.0044, 95% CI [1983, 8667]) and significantly decreased 1 h after the training (*p* = 0.0022, 95% CI [−8553, −2360]) (Figure 2b).

#### 3.1.3. Active Ghrelin Levels

In the personal training condition, compared to pre-training, the serum active ghrelin levels significantly decreased just after the training (*p* = 0.0076, 95% CI [−7.3506, −1.3732]). Compared to 15 min after the training, the serum active ghrelin level significantly decreased (*p* = 0.0054, 95% CI [−6.3586, −1.3545]). Compared to just after the training, the serum active ghrelin level significantly increased 1 h after the training (*p* = 0.0031, 95% CI [1.6654, 6.5750]). In the group training condition, compared to just after the training, the serum active ghrelin level significantly increased 1 h after the training (*p* = 0.0018, 95% CI [2.6109, 9.0825]) (Figure 2c).

#### 3.1.4. Leptin Levels

In the personal training condition, compared to pre-training, the serum leptin levels significantly decreased 15 min after and just after the training (*p* = 0.0137, 95% CI [−259.7, −35.673]; *p* = 0.0266, 95% CI [−435.7, −31.79], respectively) as shown in Figure 2d.

#### 3.1.5. Lactate Levels

In the personal training condition, compared to pre-training, the serum lactate levels significantly increased in 15 min (15 min *p* < 0.0001, 95% CI [2542.3, 4341.5]) and just after the training (*p* < 0.0001, 95% CI [2132.8, 4709.4]). Compared to 15 min after starting the exercise, the serum lactate level significantly decreased an hour after the training (*p* = 0.0005, 95% CI [−4031.8, −1450.2]) and after the training, compared to just after the training (*p* = 0.0001, 95% CI [−3798, −1642]).

In the group training condition, compared to baseline, the serum lactate level significantly increased just after and an hour after the training (*p* < 0.0001, 95% CI [2638, 4412] and p = 0.0186, 95% CI [158.29, 1450.1], respectively). Compared to just after the training, the serum lactate level significantly decreased an hour after the training (*p* < 0.0001, 95% CI [−3453.1, −1988.1]) (Figure 2e).

#### 3.1.6. DHEA-S Levels

In the personal training condition, compared to pre-training, the serum DHEA-S levels significantly increased in 15 min (*p* = 0.0195, 95% CI [0.0212, 0.2032]) and an hour after the training (*p* = 0.0351, 95% CI [0.0161, 0.3784]).

In the group training condition, compared to the baseline, the serum DHEA-S level significantly increased just after and an hour after the training (*p* = 0.0012, 95% CI [0.0997, 0.3169]; *p* = 0.0461, 95% CI [0.0023, 0.2282]). Compared to just after the training, the serum lactate level significantly decreased an hour after the training (*p* = 0.0203, 95% CI [−0.169, −0.017]) (Figure 2f).

### 3.2. Hormonal Results in Cortisol-Based Clustered Groups

Cluster analysis of the cortisol data classified participants into two groups: seven were assigned to the high cortisol group (hypersecretors, Type 1), and seven to the low cortisol group (hyposecretors, Type 2).

#### 3.2.1. Cortisol Responses in the Different Groups

**Figure 3:**
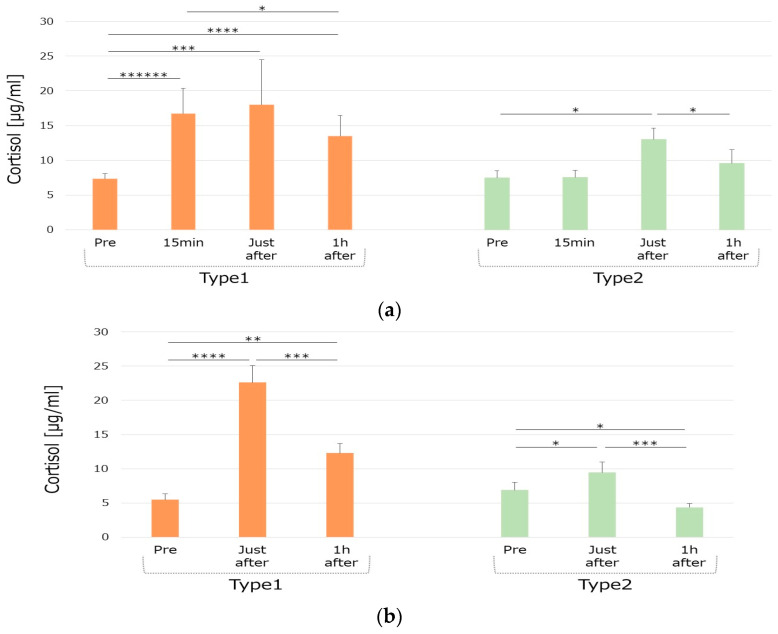
Group difference in cortisol secretion patterns under the two training conditions. (**a**): Personal training, (**b**): Group training, Type1: higher cortisol secretor, Type2: lower cortisol secretor. Significant differences are indicated as follows: * *p* < 0.05, ** *p* < 0.01, *** *p* < 0.005, **** *p* < 0.001, ****** *p* < 0.0001.

In the personal training condition for Type1 group, compared to pre-training, serum cortisol levels significantly increased in all timings (15 min *p* < 0.0001, 95% CI [6.9085, 11.743]; just after *p* = 0.0040, 95% CI [4.906, 16.431]; 1 h after *p* = 0.0005, 95% CI [3.9843, 8.40363]). Compared to 15 min after starting the experiment, the serum cortisol level significantly increased (*p* = 0.0210, 95% CI [−5.6003, −0.664]) just after the training. For Type2 group, compared to pre-training, serum cortisol levels significantly increased just after the training (*p* = 0.0220, 95% CI [1.13196, 10.0399]) and significantly decreased (*p* = 0.0449, 95% CI [−6.7898, −0.1088]) an hour after the end of the training (Figure 3a).

In the group training condition for Type1, compared to the baseline, the serum cortisol level significantly increased just after and 1 h after the training (*p* = 0.0007, 95% CI [10.5503, 23.6592]; *p* = 0.0053, 95% CI [2.92309, 10.8034]). Compared to just after the training, the serum cortisol level significantly decreased (*p* = 0.0035, 95% CI [−15.632, −4.8513]) an hour after the training. In Type2 group, compared to the baseline, the serum cortisol level significantly increased just after and an hour after the training (*p* = 0.0419, 95% CI [0.12979, 4.96079]; *p* = 0.0173, 95% CI [−4.4232, −0.6281]). Compared to just after the training, the serum cortisol level significantly decreased 1 h after the training (*p* = 0.0018, 95% CI [−7.4012, −2.7407]) (Figure 3b).

#### 3.2.2. GH Responses in the Different Groups

**Figure 4:**
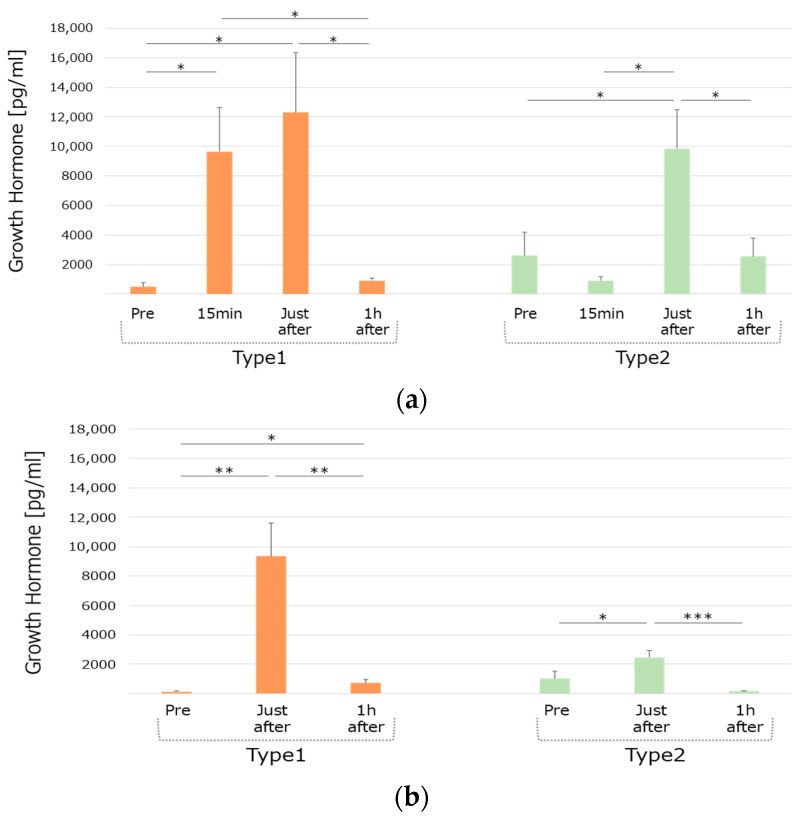
Group difference in GH secretion patterns under the two training conditions. (**a**): Personal training, (**b**): Group training, Type1: Higher cortisol secretor, Type2: Lower cortisol secretor. Significant differences are indicated as follows: * *p* < 0.05, ** *p* < 0.01, *** *p* < 0.005.

In the personal training condition for Type1, compared to pre-training, the serum GH levels significantly increased 15 min after the start of the training and just after the training (*p* = 0.0174, 95% CI [2266.5, 16,050.5]; *p* = 0.0253, 95% CI [2039.37, 21,538.1]). Compared to 15 min after the training, the serum GH level significantly increased just after the training (*p* = 0.0240, 95% CI [−15,843, −1607.7]); and compared to just after the training, the serum GH level significantly decreased 1 h after the training (*p* = 0.0284, 95% CI [−21,038, −1673.8]). For Type2, compared to pre-training, the serum cortisol levels significantly increased just after the training (*p* = 0.0485, 95% CI [66.915, 14,463]). Compared to just after the training, the serum cortisol level significantly decreased (*p* = 0.0123, 95% CI [−12,384, −2252.3]) 1 h after the end of the training (Figure 4a).

In the group training condition for Type1, compared to the baseline, the serum GH level significantly increased just after and an hour after the training (*p* = 0.0068, 95% CI [3633.5, 14,770.9]; *p* = 0.0422, 95% CI [28.473, 1136.1]). Compared to just after the training, the serum GH level significantly decreased an hour after the training (*p* = 0.0099, 95% CI [−14,300, −2940.2]). For the Type2 group, compared to the baseline, the serum GH level significantly increased just after the training (*p* = 0.0144, 95% CI [407.927, 2487.54]). Compared to just after the training, the serum GH level significantly decreased an hour after the training (*p* = 0.0016, 95% CI [−3329.8, −1255.8]) (Figure 4b).

#### 3.2.3. Other Hormonal Responses in the Different Groups

**Figure A1:**
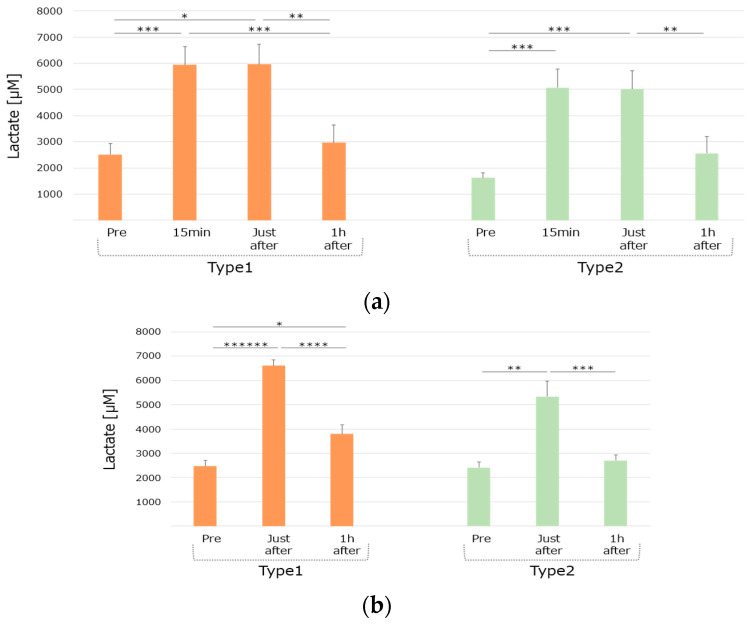
Group difference in lactate secretion patterns under the two training conditions. (**a**): Personal training. (**b**): Group training; Type1: higher cortisol secretor, Type2: lower cortisol secretor. Significant differences are indicated as follows: * *p* < 0.05, ** *p* < 0.01, *** *p* < 0.005, **** *p* < 0.001, ****** *p* < 0.0001.

**Figure A2:**
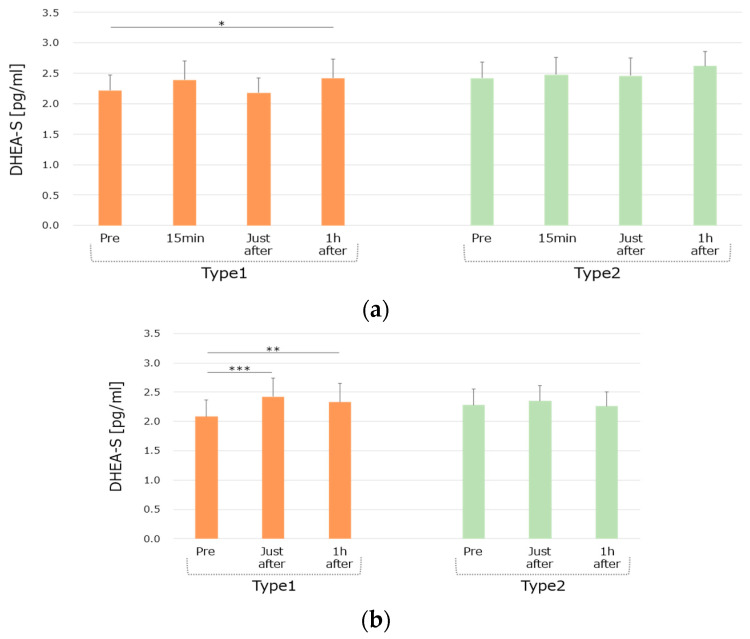
Group difference in DHEA-S secretion patterns under the two training conditions. (**a**): Personal training. (**b**): Group training; Type1: higher cortisol secretor, Type2: lower cortisol secretor. Significant differences are indicated as follows: * *p* < 0.05, ** *p* < 0.01, *** *p* < 0.005.

**Figure A3:**
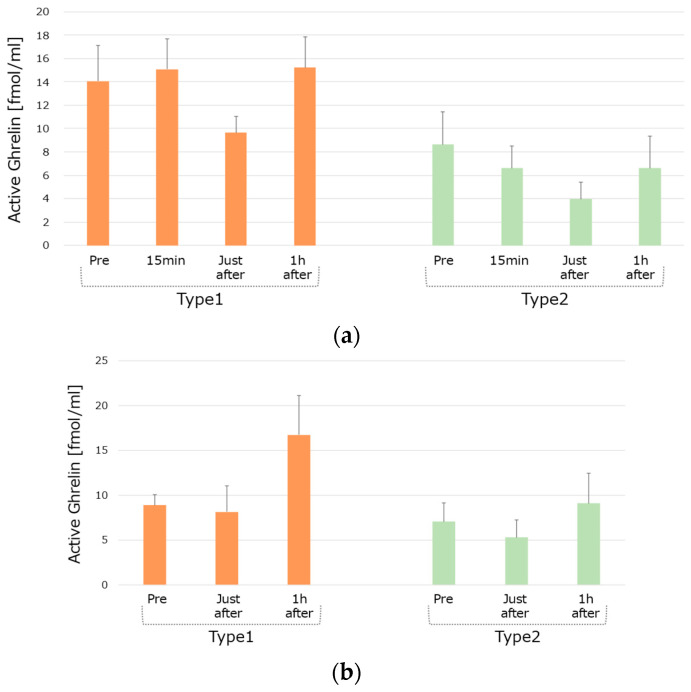
Group difference in active ghrelin secretion patterns under the two training conditions. (**a**): Personal training. (**b**): Group training; Type1: higher cortisol secretor, Type2: lower cortisol secretor. No significant differences observed.

**Figure A4:**
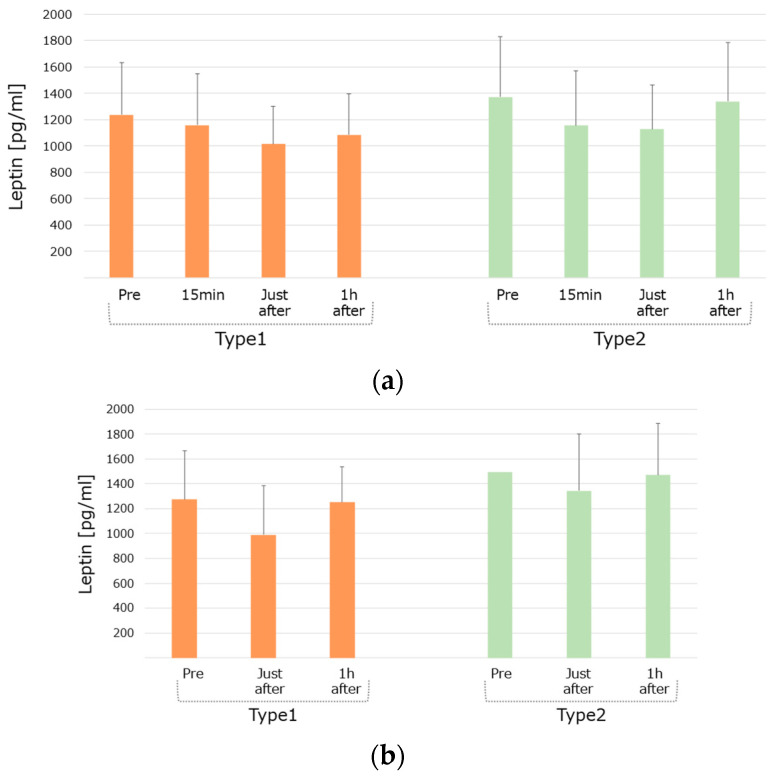
Group difference in leptin secretion patterns under the two training conditions. (**a**): Personal training. (**b**): Group training; Type1: higher cortisol secretor, Type2: lower cortisol secretor. No significant differences observed.

Lactate and DHEA-S responses for the two groups are shown in Figure A1 and Figure A2 of Appendix A, respectively. Active ghrelin and leptin responses, depicted in Figure A3 and Figure A4, respectively, did not show significant differences between the groups.

### 3.3. Questionnaire Results

#### 3.3.1. POMS-2

In the personal training condition, the confusion–bewilderment (CB) and tension–anxiety (TA) T-scores were significantly lower post-experiment compared to pre-experiment (*p* = 0.0195, 95% CI [−4.915, −0.5136] and *p* = 0.0012, 95% CI [−5.8809, −1.8334], respectively). When clustering the subjects into the two groups, only the T-score of TA in Type2 group was significantly lower post-experiment compared to pre-experiment (*p* = 0.0056, 95% CI [−8.5811, −2.2761]).

In the group training condition, the friendliness (F) scores were significantly lower post-experiment compared to pre-experiment (*p* = 0.0425, 95% CI [−5.6016, −0.1127]). However, there was no significant change in the scores when clustering the participants into two groups.

#### 3.3.2. STAI

There was no change between the pre- and post-experiment in both the personal and group training conditions, comparing data of all participants. However, Type2 participants showed significant decrease in the anxiety trait scores after the training in both the personal and group training conditions (*p* = 0.0044, 95% CI [−5.7625, −1.6661] and *p* = 0.0201, 95% CI [−4.831, −0.5976], respectively).

#### 3.3.3. NEO-PI-R

**Figure 5:**
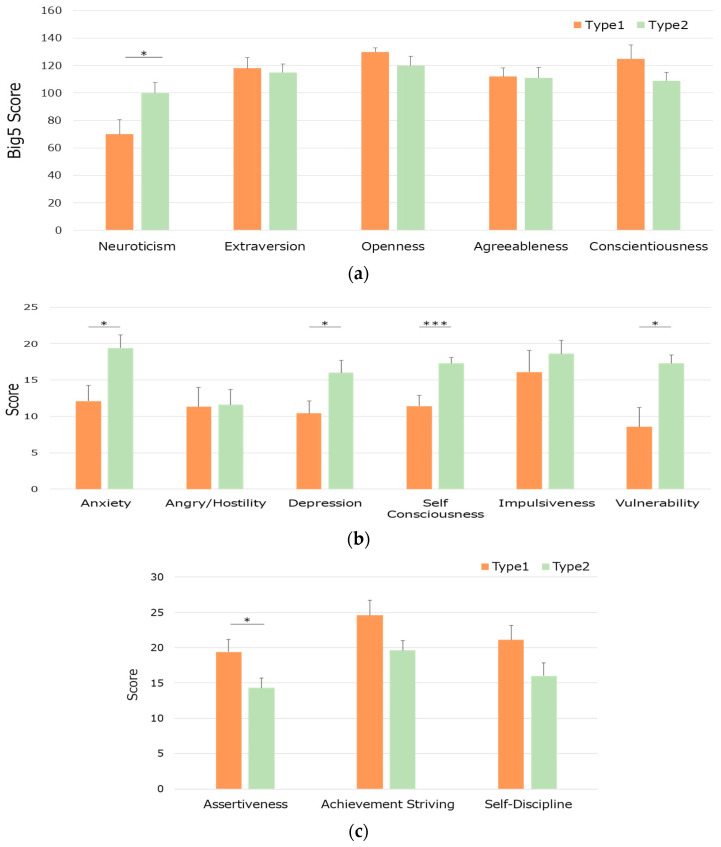
Group differences in the Big Five personality traits. (**a**): Comparison of five different dimension scores in both groups, (**b**): comparison of subscales of the neuroticism score in the two-type groups, (**c**): other different features in both groups, Type1: higher cortisol secretor, Type2: lower cortisol secretor. Significant differences are indicated as follows: * *p* < 0.05, *** *p* < 0.005.

First, we compared the five traits in both groups using Student’s *t*-test. The neuroticism score was significantly higher in the Type2 group compared to the Type1 group (*p* = 0.0374, 95% CI [2.077145, 58.20857]) (Figure 5a). Thereafter, we examined the sub-trait of neuroticism (anxiety, anger/hostility, depression, self-consciousness, impulsiveness, and vulnerability). Anxiety, depression, self-consciousness, and vulnerability scores were significantly higher in the Type2 group (*p* = 0.0237, 95% CI [1.1546, 13.417]; *p* = 0.0413, 95% CI [0.2587, 10.884]; *p* = 0.0046, 95% CI [2.1875, 9.5268]; *p* = 0.0110, 95% CI [2.3964, 15.032]) (Figure 5b).

For the other Big Five personality traits, only the assertiveness subscale of extraversion showed a significant difference with higher score in Type1 group (*p* = 0.0455, 95% CI [0.1207408, 10.16497]) (Figure 5c).

### 3.4. Pain Expression Results

**Figure 6:**
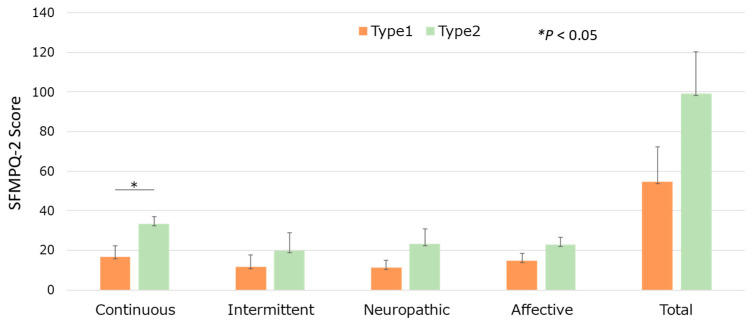
Group differences in the SFMPQ-2 score.

The participants responded to the SF-MPQ-2 to evaluate their quality of pain. The participants of the Type2 group expressed pain significantly more in the continuous component (*p* = 0.0266, 95% CI [2.3745, 30.959]). Other subscales of the SF-MPQ-2 showed no significant differences between the two groups (Figure 6).

## 4. Discussion

This study primarily aimed to investigate the endocrinological effect of exercises by assessing hormonal changes after exercise and to explore their relationship with personality traits and individual differences in pain perception/expression to experimental heat stimulation.

The increasing interest of personality psychologists lies in the role of hormones in shaping individual differences in behavior, emotion, and cognition. Hormones are chemical messengers that regulate various physiological and psychological processes in response to environmental and internal stimuli. Personality traits such as neuroticism, extraversion, openness, agreeableness, and conscientiousness can be influenced by the levels and fluctuations of hormones like cortisol, testosterone, and estrogen. Furthermore, personality traits may influence how individuals respond to hormonal changes induced by stress such as pain or social interactions with instructors. Interestingly, single individuals may have higher cortisol levels and lower testosterone levels than those in a relationship when exposed to a potential mate [31].

Personality is a complex and multidimensional construct. One of the factors that may influence personality development and expression is the endocrine system, which produces hormones that regulate various physiological and behavioral processes. Hormones are naturally occurring signaling molecules that are excreted by one part of the body and exert their influence in other parts by binding to receptors in target tissues [32,33]. Therefore, the participants’ differences in hormonal changes may be able to reflect their personality traits.

Endorphin effects are well-known by the term ‘runner’s high’, and equal amounts of adrenocorticotropic hormone (ACTH) and beta-endorphin are simultaneously produced from the precursor of beta-endorphin ACTH. ACTH also stimulates the adrenal cortex to produce cortisol, a key hormone involved in the body’s stress response. Exercise-induced changes in mood and hormonal responses, such as cortisol secretion, are well-documented and often associated with this phenomenon [9,14]. These changes are driven by endorphin release and activation of the HPA axis, leading to measurable improvements in mood and transient increases in cortisol shortly after exercise [7].

Surprisingly, Type1 and Type2 groups exhibited different patterns of hormonal secretion, especially in terms of the GH responses. A linkage has been suggested between dysfunction of the GH/IGF-1/ghrelin axis and hyperalgesia, along with several common clinical pain syndromes [34]. In this study, the Type2 group showed a relatively lower GH response level than Type1. These functional differences in hormone secretions may play an important role in personality differences, for instance, having a lower resilience with lower tolerance to pain.

The observed differences in cortisol reactivity between Type1 (higher cortisol secretors) and Type2 (lower cortisol secretors) groups suggest that hormonal responses to exercise may vary significantly based on individual physiological profiles. These findings align with prior research indicating that neuroticism influences stress reactivity and cortisol secretion [3,14]. Additionally, the variability in exercise-induced hypoalgesia (EIH) observed in our study underscores the complexity of pain modulation mechanisms.

The interplay between cortisol and other hormones, such as GH and DHEA-S, provides insights into individual differences in pain perception and personality traits. Cortisol, a key hormone in the HPA axis, regulates the body’s stress response and influences pain sensitivity [14]. Elevated cortisol levels during stress or exercise can modulate the secretion of GH, a hormone involved in recovery and metabolic regulation, by exerting catabolic effects that may inhibit the anabolic processes regulated by the GH/IGF-I axis [35]. This interaction may partly explain the lower pain tolerance observed in individuals with lower cortisol reactivity (Type2 group) compared to higher secretors (Type1 group).

DHEA-S, a hormone with neuroprotective and anti-inflammatory properties, acts as a counter-regulator to cortisol and may help buffer the impact of stress [36]. The balance between these hormones contributes to stress reactivity and pain modulation, which are further influenced by personality traits. Individuals with higher neuroticism scores often exhibit heightened cortisol reactivity, amplifying their stress sensitivity and potentially lowering their pain thresholds. These findings suggest that the dynamic interactions between cortisol, GH, and DHEA-S are critical to understanding how hormonal responses shape individual differences in personality and pain sensitivity.

The observed interplay between personality traits, hormonal responses, and pain sensitivity in this study highlights cortisol’s role as a mediator. Cortisol, a key hormone of the HPA axis, regulates stress reactivity and pain perception. Individuals in the Type 1 group, characterized by lower neuroticism scores, exhibited greater cortisol reactivity during exercise, which may contribute to lower pain sensitivity. In contrast, Type 2, with higher neuroticism scores, showed a blunted cortisol response, potentially contributing to heightened pain sensitivity. These findings align with prior research indicating that neuroticism is associated with altered HPA-axis activity and stress sensitivity, which in turn modulate pain perception [14].

The SF-MPQ-2 provided critical insights into these relationships by evaluating multiple dimensions of pain, including intensity, quality, and emotional impact. Notably, participants in the Type 2 group reported higher pain intensity in the continuous pain component compared to the Type 1 group. This aligns with the observed relationship between heightened neuroticism scores, blunted cortisol reactivity, and increased pain sensitivity [14]. The SF-MPQ-2′s multidimensional design allowed for a comprehensive assessment of pain perception, highlighting its utility in exploring the psychophysiological mechanisms linking personality traits, hormonal responses, and pain sensitivity.

Physical activity further modulates cortisol secretion and can induce EIH through endorphin release and HPA-axis activation [7,9]. In our study, individuals with lower neuroticism scores exhibited greater cortisol reactivity during exercise, which may contribute to improved pain modulation. These findings highlight the complex interactions between personality, hormonal responses, and pain sensitivity, suggesting that cortisol plays a pivotal role in these processes. Future research should explore these relationships over longer durations and across different exercise modalities.

Furthermore, cortisol reactivity and mood changes observed in this study further support the role of exercise in modulating both physiological and psychological states. Changes in mood were captured using POMS-2 and STAI scales, alongside cortisol measurements taken at three critical time points: immediately before, immediately after, and one hour post-exercise. These observations align with findings that exercise-induced cortisol release is influenced by stress responsiveness and individual differences, particularly personality traits such as neuroticism [14,37]. Individuals with higher neuroticism scores in this study exhibited distinct cortisol reactivity patterns, highlighting the interaction between personality and stress sensitivity.

While prior studies have reported consistent EIH in healthy populations [7,9,38], others have highlighted variability linked to individual factors such as fitness levels and baseline pain sensitivity [39]. Our findings add to this literature by demonstrating that personality traits and hormonal responses, such as cortisol reactivity, may influence EIH outcomes, highlighting the interplay of psychological and physiological factors.

These findings suggest that mood and hormonal responses to exercise are acute and interact with stable personality traits, providing a potential pathway for individualized exercise-based interventions. However, our results focus on short-term effects observed up to 1 h post-exercise. While previous studies suggest that regular exercise can promote long-term adaptations, such as enhanced emotional stability and stress resilience [38,39], this study does not address sustained impacts over time. Future research should employ longitudinal designs to explore whether acute responses translate into lasting changes in mood and personality traits.

Notably, distinct differences were observed between the two exercise sessions. Cortisol levels showed greater reactivity during the personal training session, likely reflecting the higher physical and mental stress of this modality compared to the group training session. In terms of psychological measures, the personal training session was associated with significant reductions in confusion–bewilderment and tension–anxiety scores, whereas the group training session showed significant changes in friendliness scores. These differences highlight the influence of exercise modality on both hormonal and psychological outcomes, underscoring the importance of context in exercise-based interventions.

While the pre-cortisol levels between the Type 1 and Type 2 groups were nearly identical, the groups differed significantly in their cortisol reactivity to exercise. Participants in the Type 1 group exhibited greater cortisol reactivity and lower pain sensitivity across both sessions. In contrast, Type 2 participants showed blunted cortisol responses and higher pain sensitivity. These findings suggest that cortisol reactivity, rather than pre-cortisol levels, plays a critical role in the interplay between hormonal responses, personality traits, and pain perception.

One of the most widely studied hormones regarding personality is cortisol, secreted by the adrenal glands in response to stress. Cortisol is involved in the regulation of the HPA axis, which is a major component of the stress response system. It has been linked to various aspects of personality, such as neuroticism, extraversion, openness, agreeableness, and conscientiousness. In common, higher levels of cortisol are associated with higher neuroticism, lower extraversion, lower openness, lower agreeableness, and lower conscientiousness [32]. However, these associations may vary depending on the context, timing, and measurement of cortisol and personality. In our study, the Type1 group showed higher cortisol levels than Type2 with a lower score of neuroticism. It has been proposed that higher basal levels of cortisol indicate stress resilience, while higher cortisol responsivity to stress may facilitate recovery in individuals sensitive to stress [14]. Higher cortisol levels during intensive training may interpret a higher resilience to stress that is not only physical but also mental.

Hormonal responses to social interactions may reveal information about personality and its adaptive functions. For instance, an investigation on the effects of psychosocial stress on hormonal responses to a social interaction with an opposite-sex individual found that relationship status and psychosocial stress moderated the relationship between an ecological cue of a potential courtship opportunity and subsequent physiological responses [31]. For example, cortisol may reflect the degree of challenge or threat that one perceives in a given situation, which may affect one’s sense of competence and autonomy. Furthermore, hormonal responses to social interactions may also reveal information about one’s motivational state and adaptive strategy for satisfying or protecting one’s basic psychological needs. Intensive training is a strong physical and mental stressor with social interaction.

The mechanisms of pain perception are multidimensional and complex, making them challenging to elucidate. Investigating individuals’ reactions to stressful situations, such as experiencing pain, by comparing baseline hormone levels with their levels in response to pain, may provide a more accessible approach to assessing this intricate topic.

### Limitations and Future Directions

While this study was carefully designed to identify individual differences in pain perception using objective hormonal measurements from blood samples, several limitations should be considered. First, the sample size (*n* = 14) was small, and the study had a gender imbalance (3 females, 11 males), which limits the generalizability of the findings. Recruitment challenges during the COVID-19 pandemic impact, combined with the intensive nature of data collection, contributed to these constraints. Future studies should aim to include larger, more diverse, and gender-balanced cohorts to enhance the robustness of the results.

Second, while cortisol and growth hormone were analyzed as key markers of hypothalamic–pituitary–adrenal (HPA) axis activity, estrogen was not included in the analysis despite its known role in modulating pain perception, particularly in females [15,37]. Including estrogen would require precise tracking of menstrual cycle phases and additional protocols, which were beyond the scope of this exploratory study. Future research should incorporate a more comprehensive hormonal profile to elucidate gender-specific mechanisms in pain sensitivity.

Third, the study did not explicitly analyze the effects of gender and age on the observed variables due to the small sample size and uneven gender distribution. However, the existing literature suggests that gender differences in pain sensitivity and hormonal responses may significantly influence results, particularly due to the impact of estrogen on the HPA axis in women [15]. Similarly, age-related changes in HPA-axis reactivity, including reduced cortisol adaptability, have been associated with altered stress and pain responses in older populations [16,17]. Future studies with larger, more diverse samples are needed to explore these relationships in detail.

Finally, the relationship between HPA-axis function evoked by intensive training tasks and pain perception cannot be conclusively determined as causal. Additionally, the study’s two-session design was focused on capturing immediate hormonal and psychological responses rather than long-term patterns. Future studies should include longitudinal designs with additional sessions to investigate the cumulative effects of exercise on hormonal responses and personality traits, as well as their influence on pain sensitivity. These studies would also help validate the hypothesis that specific hormonal reactions to exercise stimuli may serve as determinants of personality-related differences in stress reactivity and pain modulation. Advanced techniques, such as functional magnetic resonance imaging, could further elucidate these underlying mechanisms.

## 5. Conclusions

To our knowledge, this study is the first to investigate the relationship between personality traits, hormonal responses to training exercises, and individual differences in pain perception. The results suggest a potential association between personality traits, particularly neuroticism, and cortisol secretion via the HPA axis following training exercises. This indicates that personality traits may influence both stress reactivity and pain sensitivity. However, due to the exploratory nature of this study and its limited sample size, the findings should be interpreted as preliminary, emphasizing correlation rather than causation.

Additionally, individual differences in training effectiveness and motivation may be related to HPA-axis function. Repeated intensive interventions, such as personal training, have the potential to modulate hormone secretion patterns in individuals with lower training efficiency, potentially aligning them more closely with those of autonomously trained groups. This modulation could lead to a reduction in pain hypersensitivity. Future research should focus on longitudinal studies with larger and more diverse samples to confirm these associations and further explore the underlying mechanisms linking personality, hormonal responses, and pain perception.

## Data Availability

The research data supporting the reported results and conclusions of this article will be made available by the corresponding author, without undue reservation.

## Abbreviations

The following abbreviations are used in this manuscript:

ACTH	Adrenocorticotropic hormone
AH	Anger hostility
CB	Confusion bewilderment
DD	Depression dejection
DHEA-S	Dehydroepiandrosterone-Sulfate
EIH	Exercise-induced hypoalgesia
F	Friendliness
FI	Fatigue inertia
GH	Growth Hormone
HPA-axis	Hypothalamic–pituitary–adrenal axis
KAATSU	A low-intensity resistance exercise with restricted blood flow to the muscles
NEO-PI-R	NEO Personality Inventory-Revised
POMS-2	Profile of Mood States 2nd Edition
SF-MPQ-2	Short-form McGill Pain Questionnaire
STAI	State–Trait Anxiety Scale
TA	Tension anxiety
TMD	Total mood disturbance
VA	Vigor activity

## Appendix A

- Figure A1 and Figure A2 depict lactate and DHEA-S responses, respectively, for Type1 and Type2 groups under the group and personal training conditions.
- Figure A3 and Figure A4 depict active ghrelin and leptin responses, respectively, for Type1 and Type2 groups under the group and personal training conditions. Although no significant differences were found between the groups in active ghrelin and leptin, these figures are presented to provide a full overview of the study’s hormonal data.

## References

[1] Bar-Shalita T., Cermak S.A. (2020). Multi-sensory Responsiveness and Personality Traits Predict Daily Pain Sensitivity. Front. Integr. Neurosci..

[2] Vassend O., Røysamb E., Nielsen C.S. (2013). Five-factor personality traits and pain sensitivity: A twin study. Pain.

[3] Grouper H., Eisenberg E., Pud D. (2021). More insight on the role of personality traits and sensitivity to experimental pain. J. Pain Res..

[4] Naugle K.M., Fillingim R.B., Riley J.L. (2012). A meta-analytic review of the hypoalgesic effects of exercise. J. Pain.

[5] Schuch F.B., de Almeida Fleck M.P. (2013). Is Exercise an Efficacious Treatment for Depression? A Comment upon Recent Negative Findings. Front. Psychiatry.

[6] Box A.G., Feito Y., Brown C., Petruzzello S.J. (2019). Individual differences influence exercise behavior: How personality, motivation, and behavioral regulation vary among exercise mode preferences. Heliyon.

[7] Lima L.V., Abner T.S.S., Sluka K.A. (2017). Does exercise increase or decrease pain? Central mechanisms underlying these two phenomena. J. Physiol..

[8] Kami K., Tajima F., Senba E. (2017). Exercise-induced hypoalgesia: Potential mechanisms in animal models of neuropathic pain. Anat. Sci. Int..

[9] Crombie K.M., Brellenthin A.G., Hillard C.J., Koltyn K.F. (2018). Endocannabinoid and Opioid System Interactions in Exercise-Induced Hypoalgesia. Pain Med..

[10] Almagor M., Tellegen A., Waller N.G. (1995). The Big Seven model: A cross-cultural replication and further exploration of the basic dimensions of natural language trait descriptors. J. Pers. Soc. Psychol..

[11] DeYoung C.G., Mikulincer M., Shaver P.R., Cooper M.L., Larsen R.J. (2015). Openness/intellect: A dimension of personality reflecting cognitive exploration. APA Handbook of Personality and Social Psychology, Volume 4: Personality Processes and Individual Differences.

[12] Graziano W.G., Habashi M.M., Sheese B.E., Tobin R.M. (2007). Agreeableness, empathy, and helping: A person x situation perspective. J. Pers. Soc. Psychol..

[13] Bogg T., Roberts B.W. (2004). Conscientiousness and health-related behaviors: A meta-analysis of the leading behavioral contributors to mortality. Psychol. Bull..

[14] Henckens M.J.A.G., Klumpers F., Everaerd D., Kooijman S.C., van Wingen G.A., Fernández G. (2016). Interindividual differences in stress sensitivity: Basal and stress-induced cortisol levels differentially predict neural vigilance processing under stress. Soc. Cogn. Affect. Neurosci..

[15] Fillingim R.B., King C.D., Ribeiro-Dasilva M.C., Rahim-Williams B., Riley J.L. (2009). Sex, gender, and pain: A review of recent clinical and experimental findings. J. Pain.

[16] Gaffey A.E., Bergeman C.S., Clark L.A., Wirth M.M. (2016). Aging and the HPA axis: Stress and resilience in older adults. Neurosci. Biobehav. Rev..

[17] Aguilera G. (2011). HPA axis responsiveness to stress: Implications for healthy aging. Exp. Gerontol..

[18] Costa P., McCrae R. (1992). Revised NEO Personality Inventory (NEO-PI-R) and NEO Five-Factor Inventory (NEO-FFI) Professional Manual.

[19] Medoc Ltd. (2005). PATHWAY Pain & Sensory Evaluation System. [Apparatus and Software]. https://www.medoc-web.com/pathway.

[20] Dworkin R.H., Turk D.C., Revicki D.A., Harding G., Coyne K.S., Peirce-Sandner S., Bhagwat D., Everton D., Burke L.B., Cowan P. (2009). Development and initial validation of an expanded and revised version of the Short-form McGill Pain Questionnaire (SF-MPQ-2). Pain.

[21] Maruo T., Nakae A., Maeda L., Shi K., Takahashi K., Morris S., Hosomi K., Kanatani H., Matsuzaki T., Saitoh Y. (2014). Validity, reliability, and assessment sensitivity of the Japanese version of the short-form McGill pain questionnaire 2 in Japanese patients with neuropathic and non-neuropathic pain. Pain Med..

[22] Abe T., Kearns C.F., Sato Y. (2006). Muscle size and strength are increased following walk training with restricted venous blood flow from the leg muscle, Kaatsu-walk training. J. Appl. Physiol..

[23] Heuchert J.P., McNair D.M. (2012). Profile of Mood States Second Edition (POMS 2).

[24] Yokoyama K., Watanabe K. (2015). Japanese Translation of POMS 2: Profile of Mood States Second Edition.

[25] Spielberger C.D., Gorsuch R.L. (1983). Manual for the State-Trait Anxiety Inventory (Form Y) (“Self-Evaluation Questionnaire”).

[26] Hidano T., Fukuhara M., Iwawaki S., Soga S., Spielberger C.D. (2021). State Trait Anxiety Inventory (Form JYZ) Test Manual.

[27] Ward J.H. (1963). Hierarchical Grouping to Optimize an Objective Function. J. Am. Stat. Assoc..

[28] Kumari M., Badrick E., Sacker A., Kirschbaum C., Marmot M., Chandola T. (2010). Identifying patterns in cortisol secretion in an older population. Findings from the Whitehall II study. Psychoneuroendocrinology.

[29] Rasch D., Guiard V. (2004). The robustness of parametric statistical methods. Psychol. Sci..

[30] Zimmerman D.W. (1994). A note on the influence of outliers on parametric and nonparametric tests. J. Gen. Psychol..

[31] Nickels McLean N., Maestripieri D. (2023). Hormonal responses to brief social interactions: The role of psychosocial stress and relationship status. PLoS ONE.

[32] Eisenlohr-Moul T.A., Owens S.A., Zeigler-Hill V., Shackelford T.K. (2016). Hormones and Personality. Encyclopedia of Personality and Individual Differences.

[33] Depue R.A., Fu Y. (2011). Neurogenetic and experiential processes underlying major personality traits: Implications for modelling personality disorders. Int. Rev. Psychiatry.

[34] Xu J., Casserly E., Yin Y., Cheng J. (2020). A systematic review of growth hormone in pain medicine: From rodents to humans. Pain Med..

[35] Rosmond R., Björntorp P. (1998). The interactions between hypothalamic-pituitary-adrenal axis activity, testosterone, insulin-like growth factor I and abdominal obesity with metabolism and blood pressure in men. Int. J. Obes. Relat. Metab. Disord..

[36] Maninger N., Wolkowitz O.M., Reus V.I., Epel E.S., Mellon S.H. (2009). Neurobiological and neuropsychiatric effects of dehydroepiandrosterone (DHEA) and DHEA sulfate (DHEAS). Front. Neuroendocrinol..

[37] Craft R.M. (2007). Modulation of pain by estrogens. Pain.

[38] Koltyn K.F., Brellenthin A.G., Cook D.B., Sehgal N., Hillard C. (2014). Mechanisms of exercise-induced hypoalgesia. J. Pain.

[39] Fillingim R.B., Loeser J.D., Baron R., Edwards R.R. (2016). Assessment of chronic pain: Domains, methods, and mechanisms. J. Pain.

